# Relationship Between the Current Definitions of Periprocedural Myocardial Infarction and Clinical Outcomes Following Percutaneous Coronary Revascularization

**DOI:** 10.31083/RCM43839

**Published:** 2025-11-11

**Authors:** Luca Paolucci, Giulia Nardi, Marco Lombardi, Giovanni Occhipinti, Fabio Mangiacapra, Rocco Vergallo, Carmine Musto, Iginio Colaiori, Carlo Briguori, Domenico Gabrielli, Francesco De Felice

**Affiliations:** ^1^Interventional Cardiology Unit, San Camillo Forlanini Hospital, 00152 Rome, Italy; ^2^Structural Interventional Cardiology, Careggi University Hospital, 50134 Florence, Italy; ^3^Department of Internal Medicine and Medical Specialties (DIMI), Università di Genova, 16132 Genova, Italy; ^4^Cardiovascular Clinic Institute, Institut d'Investigacions Biomèdiques August Pi i Sunyer (IDIBAPS), University of Barcelona, Hospital Clínic de Barcelona, Barcelona, 08036 Catalunya, Spain; ^5^Interventional Cardiology Unit, Campus Biomedico University, 00128 Rome, Italy; ^6^Cardiothoracic and Vascular Department (DICATOV), IRCCS Ospedale Policlinico San Martino, 16132 Genova, Italy; ^7^Cardiology Unit, PO San Giovanni Evangelista, 00011 Tivoli, Italy; ^8^Interventional Cardiology Unit, Santa Maria Goretti Hospital, 04100 Latina, Italy; ^9^Interventional Cardiology Unit, Clinica Mediterranea, 80122 Naples, Italy

**Keywords:** periprocedural myocardial infarction, percutaneous coronary intervention, coronary artery disease, coronary revascularization

## Abstract

Since the beginning of the percutaneous coronary intervention (PCI) era, periprocedural myocardial infarction (PMI) has been recognized as a potential source of impaired outcomes in patients undergoing revascularization. Subsequently, several different definitions of PMI have been provided, coming from trial research groups or international consensus. Despite these efforts, the debate over the prognostic value or PMI in terms of mortality risk, as well as its role in defining composite ischemic endpoints in clinical investigations, has been extremely active. Currently, three international definitions of PMI are available: the Universal Definition of Myocardial Infarction (UDMI), the Academic Research Consortium (ARC)-2 definition, and the definition by the Society for Cardiovascular Angiography and Interventions (SCAI). These definitions differ significantly in terms of sensitivity and prognostic relevance, which has led to heterogeneous findings in clinical studies investigating this topic. Thus, this review aims to provide an overview of the main features of these definitions, their association with the risk of mortality, and how different definitions can influence the results of major investigations in the research setting.

## 1. Introduction

Recently, several studies have focused on defining the benefits and limitations of coronary revascularization in terms of major clinical outcomes [1, 2, 3, 4, 5]. Despite being effective in reducing future spontaneous myocardial infarctions (MIs) and re-interventions [6, 7], percutaneous coronary intervention (PCI) is associated with procedural risks that can limit the prognostic value of this technique in terms of mortality. Since the early stages of PCI, periprocedural MI (PMI) has been recognized as a potential source of impaired outcomes following revascularization [8]. Several studies have been conducted to evaluate the relationship between PMI and mortality; however, these studies have been characterized by heterogeneity in their design, resulting in conflicting results [6, 9, 10, 11, 12].

A significant proportion of the heterogeneous findings on this topic derives from the different definitions of PMI that have been proposed over the years. While earlier studies often relied on protocol definitions, recently, international societies have made several efforts toward standardization [13]. Thus, three internationally recognized definitions of PMI are currently available: the Fourth Universal Definition of Myocardial Infarction (UDMI), the Academic Research Consortium (ARC)-2 definition, and the definition by the Society for Cardiovascular Angiography and Interventions (SCAI) [14, 15, 16]. These definitions have subsequently been adapted through various consensus documents [17, 18] and differ significantly in terms of biomarker evaluation and the inclusion of ancillary criteria. Hence, this narrative review aims to synthesize the main features of each of the three available definitions of PMI and describe their different relationships with clinical outcomes following PCI, as per the available literature. Although PMI has significant implications in other scenarios, such as acute coronary syndromes (ACS) and cardiac surgery [19, 20], this work will primarily focus on patients with stable coronary artery disease (CAD) treated with PCI, a setting that has been most extensively investigated previously.

## 2. Main Features of the International Definitions of PMI

The three definitions of PMI discussed in this paper are reported in Table 1. Similar to spontaneous MI [14], these definitions of PMI are characterized by two components: (1) biomarker elevation; (2) ancillary criteria. To date, the only biomarkers considered for detecting myocardial damage are cardiac troponin (cTn) and cardiac-specific creatine kinase myocardial band (CK-MB). Despite the purpose of this review, it is worth noting that these two enzymes exhibit different kinetic features, which can slightly influence the detection of myocardial damage [21, 22]. In patients with normal baseline cTn values, the UDMI requires an elevation of at least 5× the upper rate limit (URL) for the used sampling assay; meanwhile, in patients with elevated baseline cTn values, a 20% variation must also be detected to define the occurrence of new myocardial damage [14]. Comparatively, the ARC-2 definition requires a cTn increase of 35× the URL, regardless of the baseline values [15]. Both these definitions also need the presence of at least one ancillary criterion to define a new PMI event. Unlike the UDMI and ARC-2 requirements, the SCAI definition of PMI primarily relies on CK-MB levels, while cTn is recommended only if the former is not available. SCAI-defined PMI requires a rise in CK-MB levels of 5× the URL or cTn of 35× the URL alongside one ancillary criterion. Alternatively, when no ancillary criteria are detected, isolated elevations in CK-MB levels of 10× the URL or cTn of 70× the URL are sufficient. These cut-offs are also valid in patients with elevated pre-procedural biomarkers [16]. Notably, the use of CK-MB has decreased recently due to the lower diagnostic accuracy of this biomarker compared to cTn, while several centers no longer have the sampling assays required to measure CK-MB [23]. This may limit the application of the SCAI definition in real-world practice. Regarding the concerns about the type of ancillary criteria, both the UDMI and ARC-2 definitions include both electrocardiogram (ECG) and non-ECG features. Conversely, only ECG alterations are used in the SCAI definition. Moreover, while the UDMI considers various subtypes of possible ECG modification, the ARC-2 and the SCAI definitions emphasize the role of new Q-waves and left bundle branch block (LBBB) development.

**Table 1.** **International definitions of periprocedural myocardial infarction**.

		UDMI	ARC-2	SCAI
Biomarker				
	cTn	5× URL	35× URL	35× or 70× URL
	CK-MB	Not recommended	Not recommended	5× URL or 10× URL*
Ancillary criteria				
	Mandatory	Yes	Yes	No
	Ischemic ECG changes	Any	New Q wave	New Q wave or LBBB
	Flow limiting angiographic complication	Yes	Yes	No
	Loss of myocardial function	Yes	Yes	No

*CK-MB to be preferred over cTn. UDMI, Universal Definition of Myocardial Infarction; ARC-2, Academic Research Consortium-2; SCAI, Society for Cardiovascular Angiography and Interventions; cTn, cardiac troponin; CK-MB, creatine kinase myocardial band; ECG, electrocardiogram; URL, upper rate limit; LBBB, left bundle branch block.

## 3. Incidence of PMI According to the Different Definitions

The prevalence of any condition, including PMI, is influenced by a balance between the sensitivity of a specific diagnostic tool (e.g., cardiac enzymes) and the criteria required to meet a definition. Notably, the diagnostic accuracy for myocardial damage has significantly increased following the introduction of high-sensitivity (HS) cTn detection, resulting in a near elimination of the risk for false negatives [24]. Consequently, the type of definition applied and the clinical context in which the investigation is conducted are the main factors influencing the rates of PMI in the literature.

Nonetheless, conflicting data have been reported regarding the rates of PMI in the most recent registries, including all-comer patients undergoing PCI. Overall, current evidence suggests that PMI occurs in 10%–15% of cases when applying the UDMI [10, 25, 26, 27, 28], 5%–10% of cases when using the ARC-2 definition [10, 25, 27, 28, 29], and in 5% or fewer patients when the SCAI definition is applied [9, 11, 25, 27, 28, 30, 31]. These trends mainly reflect the minimal biomarker elevations required to meet each one of these definitions, which are progressively higher across the UDMI, the ARC-2, and the SCAI criteria. Therefore, while the UDMI criteria can be met even with minimal cTn elevations, the SCAI definition requires more extensive cardiac damage, reducing its relative incidence. Other major influencers are represented by the complexity of coronary intervention and the amount of jeopardized myocardium involved during the revascularization, which both lead to a higher risk of PMI [32, 33]. Moreover, patient-level clinical features can independently influence the incidence of PMI, such as age, chronic kidney disease, and diabetes [34]. Ultimately, methodological issues must also be considered. Specifically, there is notable heterogeneity in the designs of previous studies in terms of routine sampling of pre- and post-PCI biomarkers [10, 11, 25, 35, 36], systematic evaluation of ancillary criteria [9, 10, 11, 26, 27, 35, 36], or the use of non-HS cTn [25, 28, 29, 31]. All these factors influence the capacity of those studies to detect PMI events and should be strictly considered when trying to infer its prevalence in patients undergoing PCI.

## 4. Relationship Between the Different Definitions of PMI and Mortality

Despite questioning the use of MI as a surrogate for all-cause and cardiovascular mortality in clinical investigations (mostly due to improvements in both interventional and medical treatment) [37], the association between spontaneous MI events and reduced survival remains relevant [38, 39]. Conversely, conflicting evidence has been published regarding the relationship between PMI and the risk of death.

For UDMI, most studies have demonstrated a 1.5- to 2.0-fold increased risk of mortality following a PMI [11, 27, 30, 40, 41, 42]. This association is progressively higher for the other two definitions, with a more than 2.0- and 3.0-fold increase for the ARC-2 [25, 27, 29, 42] and the SCAI [9, 25, 27, 29, 31, 36, 40, 42, 43] definitions, respectively. Publication bias and selective reporting of positive findings are major issues when considering the prognostic relevance of a certain event that cannot be investigated through randomized studies, since these can inflate the conclusions derived from the available literature [44]. For the specific case of PMI, the significant number of positive studies is not sufficient to rule out this risk. In a dedicated sub-analysis of the “International Study of Comparative Health Effectiveness with Medical and Invasive Approaches” (ISCHEMIA) trial, neither the primary nor the secondary protocol definitions of PMI (which relied on CK-MB and cTn, respectively) were associated with mortality [6]. Similarly, the UDMI failed to show significant prognostic value in patients randomized to PCI in the “Evaluation of XIENCE versus Coronary Artery Bypass Surgery for Effectiveness of Left Main Revascularization” (EXCEL) trial [11]. Other negative reports have been published from observational cohorts [10, 26, 28, 35]. The evidence from these investigations, combined with the risk of positive selective publishing, underscores the importance of a cautious approach when attempting to infer the mortality risk in patients experiencing PMI. Recently, we demonstrated that most of the death events reported in studies investigating this topic were of cardiac origin [42]. This supports the hypothesis that previous positive reports actually detected the presence of a true effect, but the risk that this association has been overestimated cannot be excluded.

Regarding concerns about the grade of this association, the increased prognostic relevance of the SCAI definition should support a higher threshold for biomarker elevation. Overall, a trend exists indicating an inverse relationship between each sensitivity definition and the subsequent prognostic relevance, with the UDMI and SCAI definitions being at opposite extremities of this balance [45]. Concordantly, recent studies have shown that the relative contribution of ancillary criteria to the association between PMI and mortality is progressively lower or even negligible when higher myocardial damage is identified, as indicated by biomarker sampling [27, 42]. To date, whether a risk stratification model based solely on objective measurements of myocardial damage can be superior to traditional PMI definitions remains unclear. Despite some investigations demonstrating the reliability of this approach even in complex settings, such as acute coronary syndromes [19], others have reported that similar biomarker elevations in the context of PMI or spontaneous MI are associated with different prognostic relevance, suggesting that other factors are surely involved in the final mortality risk [46].

## 5. The Role of PMI Definitions in the Composite Outcomes of Clinical Trials

With the introduction of composite outcomes in cardiovascular research, the statistical power of trials investigating different aspects of coronary revascularization has become dependent on the relative rates of other clinical events, including MI [47]. Considering the major differences in incidence between these definitions, the choice of a specific PMI definition can lead to significant changes in the results of a certain study. While more sensitive definitions (such as the UDMI) can be useful for detecting even minor events and increasing the statistical power of a particular trial, the relatively limited association of these definitions with mortality raises questions about their prognostic relevance [45, 48]. Opposite considerations could be applied to less sensitive definitions, such as the SCAI definition. This has been elegantly documented by Spitzer *et al*. [49], who reported how the MI events are by far the ones most exposed to heterogeneous definitions across contemporary coronary intervention trials.

Several examples of the complex heterogeneity resulting from these different definitions can be proposed. Within trials comparing PCI against coronary artery bypass grafting (CABG) in left main or multivessel disease, protocol-defined PMI [50, 51, 52], the SCAI definition [53, 54], the UDMI [55], or no definition [56] have all been used previously [57]. The influence of each definition on the overall findings of those studies has been largely debated. In a renowned editorial by Serruys *et al*. [57], the authors demonstrated how the application of different ways to define PMI could dramatically change the 5-year results of the “TAXUS Drug-Eluting Stent Versus Coronary Artery Bypass Surgery for the Treatment of Narrowed Arteries” (SYNTAX) trial. Specifically, when the protocol definition or the UDMI was applied, the composite endpoint of major cardiac and cerebrovascular events was reduced by a CABG strategy. On the other hand, when the SCAI definition was used, PCI appeared not to be inferior to a surgical approach [57]. Resembling results were reported in a pooled analysis of trials comparing PCI and CABG in patients with left main disease [58]. Notably, this issue is not limited to studies comparing different revascularization strategies; similar heterogeneities can also be found in trials investigating medical therapy versus revascularization [1, 4, 59], intracoronary physiology [60, 61, 62], intracoronary imaging [63, 64, 65, 66], and other areas [67]. The debate on how to define PMI events in major clinical trials has been active for several years. While the use of more prognostically relevant definitions may result in a more appealing approach to detect larger MI with a higher risk of mortality [45, 46], evidence has been published showing that even minor myocardial damage can have a clinical value [42]. Alternative solutions to the rigid application of one specific definition have been proposed. In the ISCHEMIA trial, two different definitions of PMI were contemporarily applied [4, 6]. A similar approach has been used in a post hoc analysis of the EXCEL trial [11]. This strategy can facilitate different interpretations of the study findings, thereby reducing the risk of drawing misleading conclusions based on pre-specified outcome definitions. To perform such analyses, the routine and standardized collection of post-PCI biomarker elevation (ideally two samplings at 8 h and 16 h) together with the systematic assessment of ancillary criteria within the first 48 hours is mandatory [49]. A significant example in this regard can be provided by the three “A Clinical Trial Comparing Cangrelor to Clopidogrel Standard Therapy in Subjects Who Require Percutaneous Coronary Intervention” (CHAMPION) studies, which compared P2Y12 inhibition with cangrelor against clopidogrel following PCI in different clinical settings [68, 69, 70]. While the two earlier trials relied on a protocol-defined PMI (CHAMPION-PCI and CHAMPION-PLATFORM), the CHAMPION-PHOENIX trial implemented the UDMI criteria. Despite this major difference, the routine assessment of post-PCI biomarker elevations and ancillary criteria allowed for homogenizing the PMI definition on the UDMI in a subsequent pooled analysis of those three studies, which showed that cangrelor could actually be associated with a periprocedural benefit in terms of ischemic events [67].

Another possible strategy to overcome the limitations associated with a rigid application of a single PMI definition may be the use of hierarchical/weighted composite endpoints, which are designed to tailor the results of a specific study according to the clinical relevance of each isolated component of the primary outcome. These techniques are becoming increasingly popular in cardiovascular research, primarily because they preserve the benefits of composite endpoints in terms of study power while providing prognostically robust results [71]. In the case of PMI, it has been proposed to locate such events immediately below spontaneous MI [72]. Despite their appeal, these outcomes have been utilized in only a few coronary intervention studies, primarily as retrospective analyses of previously published trials. Therefore, it remains unclear whether hierarchical/weighted outcomes will replace classical time-to-event composite endpoints in the future [73, 74].

## 6. Future Perspectives

The quest for the perfect definition of PMI has been a years-long journey, which has led to the generation of several international recommendations and significant debate within the cardiovascular scientific community [45, 49, 57, 75]. Each one of the available PMI definitions shows specific strengths and limitations, not only in terms of the ratio between sensitivity and prognostic power, but also in terms of generalizability in complex settings, such as acute coronary syndromes and CABG (Table 2) [15, 42]. While a definition of PMI is surely needed when conducting a specific investigation, it is our opinion that those limitations should be strictly accounted for. Considering that the UDMI, ARC-2, and SCAI definitions encompass a broad spectrum of biomarker elevations and subtypes of ancillary criteria, we believe it is unlikely that a revised definition of PMI could overcome the limitations found in the existing ones. If we accept the hypothesis that including MI events in the composite endpoints of clinical trials is a way to infer cardiac mortality risk, then we should also acknowledge that this relationship is not dichotomous but reflects a continuous association between the amount of cardiac damage and the consequent prognosis. Unlike spontaneous MI, whose objective quantification can be hindered by non-predictable factors, such as the time comprised between myocardial ischemia and coronary revascularization [76], PMI can only occur in a fully controllable setting. Previous studies have suggested that a detailed collection of biomarkers following coronary revascularization can provide an accurate representation of the subsequent mortality risk [20, 27, 77, 78, 79, 80]. Accordingly, a detailed depiction of post-PCI myocardial damage based on reporting objective cTn/CK-MB elevations at specific time points could be more informative compared to the use of a dichotomic outcome. This approach could provide a quantitative and continuous assessment of these adverse events, allowing for tailored future analyses. Eventually, if combined with routine evaluation of ancillary criteria, a similar strategy could enable a global estimation of the clinical risk, independent of the limitations of a specific definition of PMI. Despite this, it is worth noting that a systematic assessment of post-PCI myocardial injury in clinical practice would require significant efforts in terms of patient care, time, and costs. Moreover, the use of multiple assays to estimate cardiac biomarkers could limit the comparability of sampling data obtained from various centers in a hypothetical research setting. Presently, no studies have been conducted to evaluate the feasibility of this approach and to compare it with the strategies traditionally used to detect PMI. Therefore, this hypothesis should be considered only speculative, and dedicated investigations are needed to address the clinical and research validity of the hypothesis.

**Table 2.** **Main strengths and limitations of the current international definitions of PMI**.

	Strengths	Limitations
UDMI	(1) High sensitivity	(1) Low prognostic value
	(2) Simulate the international definition for spontaneous MI	(2) Alternative definition for patients with ACS and undergoing CABG
	(3) Largely investigated in major studies	(3) Relying on both biomarkers and ancillary criteria evaluation
ARC-2	(1) Moderate sensitivity and prognostic value	(1) Rarely investigated in major studies
	(2) Do not provide alternative definitions for patients with ACS or undergoing CABG	(2) Relying on both biomarkers and ancillary criteria evaluation
SCAI	(1) High prognostic value	(1) Low sensitivity
	(2) Provide an alternate definition relying solely on biomarker evaluation	
	(3) Largely investigated in major studies	
	(4) Do not provide alternative definitions for patients with ACS or undergoing CABG	

MI, myocardial infarction; ACS, acute coronary syndromes; CABG, coronary artery bypass grafting.

## 7. Conclusions

The three current international definitions of post-PCI PMI dramatically differ in terms of sensitivity and prognostic relevance. Therefore, this narrative review aimed to synthesize the main strengths and limitations of these studies, as well as their potential influence on the results obtained from the available literature. Notably, the PMI definitions have not remained constant over the years, but rather reflect the ongoing efforts of scientific societies to address the clinical and research needs of the cardiovascular community. While future dedicated studies are needed to address this topic, a dynamic and comprehensive approach to estimating post-PCI myocardial damage should be recommended to improve the risk stratification of these patients in real-world practice [13].

This review has several limitations that should be taken into consideration. Due to its narrative nature, the lack of systematic research on studies investigating the relationship between PMI and adverse events may have influenced the conclusions of our work. Moreover, the quality of the reported studies has not been critically evaluated using standardized methods. This, together with the significant heterogeneity of those reports and their mostly observational nature, limits the generalizability of those findings. Ultimately, the reported perspectives and conclusions on this topic reflect the opinions of the authors, which are not supported by dedicated research.
